# Retraction: Heterotypic neutrophil-in-tumor structure: a novel pathological feature first discovered in the tissues of OPSCC

**DOI:** 10.3389/fonc.2025.1699151

**Published:** 2025-09-11

**Authors:** Frontiers Editorial Office

Following publication, concerns were raised regarding the validity of the data in the article after the authors submitted a correction. The authors failed to provide the raw data or a satisfactory explanation during the investigation, which was conducted in accordance with Frontiers’ policies. Given the concerns, and the lack of raw data, the editors no longer have confidence in the findings presented in the article.

This retraction was approved by the Chief Editors of Frontiers in Oncology and the Chief Executive Editor of Frontiers. The authors received a communication regarding the retraction and had a chance to respond. This communication has been recorded by the publisher.

